# Effect of perceptive-auditory training on the classification of speech hypernasality

**DOI:** 10.1590/2317-1782/20232022069en

**Published:** 2023-09-15

**Authors:** Flora Taube Manicardi, Jeniffer de Cássia Rillo Dutka, Thais Alves Guerra, Maria Inês Pegoraro-Krook, Eduardo Federighi Baisi Chagas, Viviane Cristina de Castro Marino

**Affiliations:** 1 Programa de Pós-graduação em Fonoaudiologia, Universidade Estadual Paulista Júlio de Mesquita Filho - UNESP - Marília (SP), Brasil.; 2 Pós-graduação em Ciência da Reabilidação, Hospital de Reabilitação de Anomalias Craniofaciais, Universidade de São Paulo - USP -Bauru (SP), Brasil.; 3 Departamento de Educação Física, Nutrição e Fisioterapia, Universidade de Marília - Unimar - Marília (SP), Brasil.

**Keywords:** Cleft Palate, Velopharyngeal Insufficiency, Speech Disorders, Speech Perception, Speech

## Abstract

**Purpose:**

To analyze the effect of auditory-perceptual training by inexperienced speech-language pathologists in the classification of hypernasality in individuals with cleft lip and palate and compare their classification of hypernasality individually, with the gold standard evaluation, before and after this training.

**Methods:**

Three inexperienced speech-language pathologists used a four-point scale to assess 24 high-pressure speech samples from individuals with cleft lip and palate, before and after auditory-perceptual training. The speech samples corresponded to six samples of each degree of hypernasality. The speech-language pathologists received auditory-perceptual training during the assessments. They had access to anchor samples and immediate feedback of correct answers regarding the degree of hypernasality in training.

**Results:**

There was no significant difference in the overall percentage of correct answers when comparing before and after the auditory-perceptual training. There was a significant association and agreement of the three evaluators with a gold standard evaluation after training, with an increase in agreement for a single evaluator for absent and mild degrees of hypernasality. The dichotomous analysis of the data showed an increase in the Kappa Index of Agreement of this evaluator. Although there was an increase in the Index of Agreement between evaluators for absent, mild, and severe hypernasality, this increase did not reach statistical significance.

**Conclusion:**

The auditory-perceptual training provided did not result in a significant improvement in the hypernasality classification for the inexperienced speech-language pathologists, even though the individual data analysis showed that the training favored one of the evaluators. Further studies involving gradual and more extensive auditory-perceptual training may favor the classification of hypernasality by inexperienced SLPs.

## INTRODUCTION

Hypernasality is the most representative speech symptom of velopharyngeal dysfunction (VPD) after primary palatal surgery^(1)^. This excessive nasal resonance during the production of oral sounds^(1)^ occurs due to abnormal coupling of the resonance cavities (oral and nasal). Although instrumental techniques (nasoendoscopy, videofluoroscopy and flow-pressure technique) are recommended to corroborate the diagnostic of VPD, the auditory-perceptual assessment is the initial tool used by clinicians to identify speech symptoms suggestive of VPD after primary palatoplasty^(2,3)^. Through their auditory impressions, the clinicians identify the presence and severity of hypernasality, which favor clinical decision-making and evaluation of the effectiveness of the treatment^(4)^. The auditory-perceptual evaluation must be conducted by experienced professionals to minimize variations and errors inherent to the subjective nature of this type of evaluation^(5)^.

The identification of hypernasality can be challenging even for experienced listeners^(6,7)^. The type of stimuli (speech or song)^(8)^, the extent of the stimulus, the phonetic context of the speech sample^(1,9,10)^ and the presence of coexisting alterations^(11)^ can influence perceptual assessment of hypernasality, affecting its reliability^(4)^. The type of scale^(4,12-14)^ and the listener's familiarity with the use of a given scale can also influence the assessment of this speech symptom^(13)^. The numerical scale of equal intervals is widely used for clinical^(15)^ and research purposes^(6,10,16-18)^, including those involving auditory-perceptual training for the classification of hypernasality^(19,20)^.

The degree of clinical experience and the criteria adopted by each evaluator in their analyses can also affect the classification of hypernasality^(4)^. The auditory-perceptual training and the use of reference samples are strategies that can favor the consistency of the evaluators' analyses^(6)^, in which unstable internal patterns of the listeners can be replaced by references^(4)^. Although these strategies are strongly recommended to improve the reliability of the auditory-perceptual analysis of hypernasality^(6,21,)^ only few studies documented the results of auditory-perceptual training in the assessment of this speech symptom^(6,19-21)^.

In a previous study^(6)^, controlled training and definition of standardized criteria for the classification of hypernasality led to an increasing agreement within and between experienced evaluators (SLPs) and, consequently, an improvement in the reliability of the auditory-perceptual analysis of this speech symptom. Other studies particularly investigated the influence of the auditory-perceptual training in hypernasality judgments performed by students in speech-language pathology^(20,22)^ or residents in otorhinolaryngology^(19)^.

Lee, Whitehill and Ciocca^(22)^ studied the effect of practice and feedback on intra-judge and inter-judge reliability of hypernasality judgements performed by SLP-students. Significant differences were found between (1) the groups receiving training (practice with and without feedback) and (2) the group that had simple exposure to speech samples. Groups that had received more comprehensive training had a significantly larger range of hypernasality ratings than the exposure group. There were no significant differences in intra-judge reliability among the three listener groups (practice with feedback, practice without feedback and only exposure to samples). The researchers attributed this result to the small speech sample size (82 speech samples that were divided in 4 training steps) and limited training length (30 minutes of the introduction and one hour in each step of the training), which may have influenced the listeners to achieve good reliability scores.

In a later study, Guerra^(20)^ studied ratings of hypernasality performed by undergraduate SLP-students. The ratings were made at different times (before training, immediately after training, one week and one month after training) and four different conditions (without training, with training and optional access to reference samples; with training and controlled access to reference samples; and, with training, controlled access to reference samples and immediate feedback of right answer). Even controlling the possible impact of articulatory alterations in the classification of hypernasality by using only low intraoral pressure speech samples, no significant difference was not found in all hypernasality ratings among all conditions and different analysis time.

Sydney et al.^(19)^ determined the ability of otolaryngology residents (control group vs. training group) to rate hypernasality in patients with VPD. Although there was an improvement in agreement after training, this difference was not significant.

There is a need for better understanding of the ability of inexperienced SLPs in rating hypernasality as well as the strategies that may improve their initial ratings. Previous findings^(20,22)^ of auditory-perceptual judgments of hypernasality carried out by undergraduate students in SLP are inconsistent in the current literature. In addition, for otolaryngology residents, there was a trend towards improvement in the agreement of ratings after training, but with no significant difference^(19)^. In this sense, it is not clear whether a controlled auditory-perceptual training can influence the classification of hypernasality by listeners with limited clinical experience in the field of speech pathology.

The aims of this study were: 1) analyze the effect of auditory-perceptual training of inexperienced SLPs in the classification of hypernasality of individuals with CLP; 2) compare ratings of presence and degree of hypernasality of each inexperienced listener with the gold standard assessment, before and after the auditory-perceptual training.

## METHODS

This study was approved by the Research Ethics Committee at the Hospital de Reabilitação de Anomalias Craniofaciais - Universidade de São Paulo, in Bauru, SP Brazil (No. 3.131.704.3.131.704). All participants who agreed to participate signed informed consent.

Pre-existing speech recordings from individuals with a history of cleft lip and palate (CLP), both genders, were selected for this study. The pre-existing speech recordings selected were further evaluated for three participants under two conditions: before and after auditory-perceptual training.

### 2.1 Speech samples

The speech recordings were retrieved from a pre-existing set of recordings saved into computer files. These recordings were originally obtained in an acoustically treated room and had good audio quality. The speech material was recorded directly on a computer with a Sound Blaster Audigy 2 sound card and Sony® Sound Forge v8.0, with a sampling rate of 44100 Hz, in single channel, 16 Bit. The audio signal was captured using a headset microphone (AKG C420®). All pre-existing recordings had a standardized interval of one second between each phrase. The speech stimuli of the study consisted of a set of 12 high-pressure consonant phrases, in which each phrase comprised a single recurring pressure consonant target sound. This stimuli was selected considering findings of a previous study, in which perceptual ratings of hypernasality were more favorable for extended oral stimuli, even with the presence of other speech disorders.

The speech samples selected for this study comprised forty-eight samples (in audio) stored in computer files that represent the hypernasality degrees: absent (A), mild (MI), moderate (MO) and severe (S), in male and female voices. These samples consisted of a set of 12 oral high-pressure phrases. For selection purposes, all the speech samples of the databases were listened to and the first 12 samples of these bases that represent each of the 4 hypernasality degrees were included, in total 48 speech samples. The representativeness of the recorded speech samples regarding the four degrees of the scale (A=25%; MI=25%; MO=25%; and S=25%) was established in a previous study^(23)^.

In Silva-Mori´s previous study^(23)^, three Brazilian certified-SLPs with at least 10 years’ experience working in a large craniofacial center and with daily clinical routine in identifying speech disorders in individuals with CLP (Specialists SLP) achieved 100% agreement in their ratings. Therefore, for this study, the specialists’ ratings of 48 speech samples were used as the gold standard for comparisons with the participants’ ratings and also to select speech samples for auditory perceptual training. The samples included in this study were controlled for dysphonia. Other speech symptoms (nasal air emission, compensatory articulation) were not controlled.

The 48 speech samples representing the four degrees of hypernasality initially selected for the study were subsequently distributed into rating samples, reference, training and retraining samples. Thus, the 48 samples were distributed into 24 classification samples (evaluators' analysis, before and after training), 8 reference samples (anchor), 8 auditory-perceptual initial training samples and 8 retraining samples. In turn, the 24 classification samples consisted of 6 representative samples of each 4 degrees of hypernasality (6 A, 6 MI, 6 MO, 6 S) paired by gender. Finally, the 8 reference samples, 8 training samples and 8 retraining samples consisted of 2 samples of each degree of hypernasality (2A, 2MI, 2MO, 2S) also paired by gender.

### Participants

Three female certified-SLPs, mean age of 24 years, native speakers of Brazilian Portuguese were invited as participants for this study. All of them were starting the residence ship in the craniofacial area. All participants self-reported normal hearing and no previous experience in classifying hypernasality degrees in individuals with CLP.

### Procedures

The participants assessed the 24 classification speech samples (6A, 6MI, 6MO, 6S) using a 4-point scale, in two moments: before and after perceptual-auditory training. Samples were presented simultaneously by the first author (FTM) through a Powerplay PRO-8 HA8000 signal divider and individual high-quality headphones (AKG Harman, model K414P). The answers were inserted on an Excel^®^ spreadsheet.

Samples could be heard as many times as the participant deemed necessary to carry out their judgment. When necessary, the participants could ask for the repetition of the sample to the first author and the other participants could choose if they would listen to the sample again or not. Other speech symptoms (nasal air emission, compensatory articulation) were not controlled. Only samples with normal voice were included in this study.

#### Auditory-perceptual training

The auditory-perceptual training was performed by a Powerpoint presentation, which consisted of a calibration session, training and retraining. In the calibration session, the participants were introduced to the concept of hypernasality and were instructed to use the 4-point scale to rate the degrees of hypernasality. Three recorded audio speech samples (which were not included in any other stage of the study) were presented to the participants so that they could become familiar with the task.

In person auditory-perceptual training and retraining sessions were offered by the first author, for each participant individually, using a high-quality headphone (AKG Harman, model K414P) and a computer to indicate their answers on an Excel^®^ spreadsheet.

For the auditory-perceptual training itself eight references (anchor samples), were four voice males and four voice females, representing each degree of hypernasality (A, MI, MO, S) and presented to each participant. Participants were instructed to rate hypernasality using a 4-point scale. After each answer, the evaluator provided feedback regarding the degree of hypernasality. If the participant rated the sample incorrectly, then the evaluator re-presented two reference samples selected for this study. One reference sample corresponded to the correct rating degree of hypernasality. The other corresponded to the previous evaluator´s rating, that is, the sample rated incorrectly. Then, the evaluator presented the target sample again only for participant´s comparison with reference samples. After every 20 minutes of training, a five-minute break was performed in order to avoid fatigue.

Retraining was carried out one week after the auditory-perceptual training, using 8 different speech samples from those used in the training. Reference samples (N=8) used in the auditory-perceptual training were also used as references during ratings of the retraining samples. The same procedures used in the auditory-perceptual training were adopted in the retraining with a single difference: the samples were presented at random in order to prepare the participants for the final classification step.

### Data analysis

Quantitative variables were described by the mean and standard deviation (SD). The normality distribution of the percentage of correct answers was verified by the Kolmogorov-Smirnov test. The Wilcoxon signed-rank test was used to compare the mean percentage of participants' correct answers in relation to the gold standard evaluation, before and after the auditory-perceptual training.

Qualitative variables were described by the absolute frequency distribution. Chi-squared test was used to assess the association between qualitative variables (answers from each participant versus gold standard evaluation), taking into account the degrees of hypernasality. Chi-squared test was also used to further assess the association between qualitative variables for presence and absence of hypernasality (dichotomous analysis), both before and after the auditory-perceptual training.

The Cohen's kappa coefficient (k) was used to analyze the agreement between each participant and the gold standard evaluation and the moment. For the strength of agreement associated with Kappa statistics, the following labels were assigned to the corresponding Kappa intervals: <0.00 No agreement (N); 0.00 - 0.19 Slight (S); 0.20 - 0.39 Fair (F); 0.40 - 0.59 Moderate (M); 0.60-0.79 Substantial (SB) and 0.80-1.00 Almost perfect (AP). The difference between moments (before and after training) for k was analyzed by 95% confidence intervals. The SPSS v19.0 for Windows was used for all statistical analyses, adopting a significance level of 5% (p<0.05).

## RESULTS

The mean percentage of correct answers in rating the degree of hypernasality of the three participants (evaluators), before and after the auditory-perceptual training, was 65.3% and 62.5%, respectively. There was no significant difference in the overall percentage of correct answers when comparing training moments (*p-value*=0.972; Wilcoxon signed-rank test).

Additionally, before training, a significant association was observed between the three evaluators (EV1, EV2 and EV3) with the gold standard evaluation. EV1 showed moderate agreement, EV2 substantial agreement and EV3 regular agreement (Table 1). After training, there was a significant association and agreement of the three evaluators with the gold standard evaluation. EV1 continued to show moderate agreement, EV2 had a reduction in agreement in relation to the pre-training moment and EV3 showed improvement in agreement (Table 2).

**Table 1:** Analysis of the agreement and association of the absolute frequency distribution of the classification of the hypernasality of each evaluator with the gold standard rating, before the auditory-perceptual training, by degree of hypernasality^(1)^

Before training		Gold standard rating					*X^2^*	Kappa
		Absent	Mild	Moderate	Severe		p-value	
EV 1	Absent	**6**	2	0	0		<0.001*	0.556‡
	Mild	0	**4**	3	0			
	Moderate	0	0	**3**	3			
	Severe	0	0	0	**3**			
EV 2	Absent	**6**	2	0	0		<0.001*	0.667‡
	Mild	0	**3**	1	1			
	Moderate	0	0	**5**	1			
	Severe	0	1	0	**4**			
EV 3	Absent	**3**	2	0	0		<0.001*	0.389
	Mild	3	**2**	1	1			
	Moderate	0	2	**5**	2			
	Severe	0	0	0	**3**			

* significant association by the Chi-square test for p-value ≤0.05;

‡ Significant kappa coefficient for p-value ≤0.05. Findings in bold signify results that agree with the gold standard rating

**Caption:** EV=evaluator

**Table 2:** Analysis of the agreement and association of the absolute frequency distribution of the evaluation of the hypernasality classification of each evaluator with the gold standard evaluation, after auditory-perceptual training, by degree of hypernasality^(2)^

Before training		Gold standard evaluation					*X^2^*	Kappa
		Absent	Mild	Moderate	Severe		p-value	*K*
EV 1	Absent	**6**	1	0	1		<0.001*	0.500‡
	Mild	0	**5**	2	0			
	Moderate	0	0	**2**	3			
	Severe	0	0	2	**2**			
EV 2	Absent	**6**	2	0	0		<0.001*	0.333‡
	Mild	0	**3**	3	2			
	Moderate	0	1	**1**	2			
	Severe	0	0	2	**2**			
EV 3	Absent	**6**	1	0	0		<0.001*	0.667‡
	Mild	0	**5**	1	1			
	Moderate	0	0	**4**	2			
	Severe	0	0	1	**3**			

* significant association by the Chi-square test for p-value ≤0.05;

‡ Significant kappa coefficient for p-value ≤0.05. Findings in bold signify results that agree with the gold standard evaluation

**Caption:** EV=evaluator

Dichotomous analysis (presence and absence of hypernasality) further showed that all the evaluators presented a significant association and significant agreement with the gold standard evaluation before the auditory-perceptual training. However, EV3 showed an increase in the Kappa Index of Agreement with the gold standard evaluation after the auditory-perceptual training (Table 3).

**Table 3:** Comparison of the results of the participants (evaluators) with the gold standard rating, before and after the auditory-perceptual training, according to the dichotomous analysis^(24)^

		Gold standard rating		*X^2^* *p value*	Kappa
		Present	Absent		
Before training					
EV 1	Present	**16**	0	<0.001*	0.800‡
	Absent	2	6		
EV 2	Present	**16**	0	<0.001*	0.800‡
	Absent	2	6		
EV 3	Present	**16**	3	0.047*	0.412‡
	Absent	2	3		
After training					
EV1	Present	**16**	0	<0.001*	0.800‡
	Absent	2	6		
EV 2	Present	**16**	0	<0.001*	0.800‡
	Absent	2	6		
EV 3	Present	**17**	0	<0.001*	0.895‡
	Absent	1	6		

* significant association by the Chi-square test for p-value ≤0.05;

‡ Significant kappa coefficient for p-value ≤0.05

**Caption:** EV=evaluator

Table 4 shows the results of the analysis of agreement between the evaluators, before and after the auditory-perceptual training, by each degree of hypernasality and total ratings. Although there was significant agreement in the Cohen's kappa coefficient before the auditory-perceptual training among the three evaluators for the absent, moderate and severe degrees and total ratings, there was no agreement among the evaluators for mild hypernasality. Additionally, there was significant agreement in the Cohen's kappa coefficient after the auditory-perceptual training among the three evaluators for the absent, mild and severe degrees and in total ratings, but there was no agreement among the evaluators for moderate hypernasality. When comparing the findings obtained before and after the auditory-perceptual training, an increase in the Index of Agreement was observed for absent, mild, and severe hypernasality and total ratings. However, this increase was not statistically significant (analysis of confidence interval of 95%).

**Table 4:** Agreement between raters (Kappa coefficient) before and after auditory-perceptual training by response category and for total agreement^(3)^

Training	Categories	Kappa	IC 95%		p-value
			LI	LS	
Before	absent hypernasality	0.597	0.366	0.828	<0.001*
	mild hypernasality	-0.072	-0.303	0.158	0.538
	moderate hypernasality	0.261	0.030	0.491	0.027*
	severe hypernasality	0.356	0.125	0.587	0.003*
	Total	0.284	0.147	0.420	<0.001*
After	absent hypernasality	0.681	0.450	0.912	<0.001*
	mild hypernasality	0.280	0.049	0.511	0.0018*
	moderate hypernasality	0.074	-0.157	0.305	0.532
	severe hypernasality	0.600	0.369	0.831	<0.001*
	Total	0.413	0.276	0.550	<0.001*

* significant kappa coefficient for p-valor ≤0.05

**Caption:** IC = Confidence Interval; LI = Upper Limit; LS = Inferior Limit

## DISCUSSION

This study investigated the effect of auditory-perceptual training of SLPs with no experience in rating speech hypernasality in individuals with CLP. The results obtained in the present study agree with those reported by the study of Sydney et al.^(19)^, that showed that training did not affect the rating task by participants.

Findings of this study are contrary to the findings obtained by Lee et al.^(22)^. These authors found a significant difference in hypernasality ratings for the groups that received training (with or without feedback) and the group that was only exposed to speech samples. Although the present study offered training and retraining, both with feedback for correct answers, the focus was only the rating of hypernasality degrees, regardless of whether this symptom occurred in coexistence with other speech disorders. Lee et al.^(22)^, on the other hand, offered a gradual training, in a hierarchy from simpler to more difficult tasks, which may have favored the assessment of hypernasality even in the coexistence of other speech disorders.

The findings of the present study agreed with the results obtained previously by Guerra^(20)^. Some researchers argue that rating the degree of hypernasality can be even more challenging with the presence of multiple speech disorders due to the need to accurately isolate individual auditory perceptual characteristics^(25)^. Guerra^(20)^ controlled the possible impact of compensatory articulations on hypernasality ratings performed by SLP-students by including only low intraoral pressure sounds in the samples rated. The present study controlled only the voice disorders and suggested new training`s proposal. The amplified listener`s training is recommended for future studies to make them capable of identifying other speech`s disorders with or without hypernasality. This study also analyzed the association between qualitative variables (answers from each participant versus gold standard ratings), considering the degrees of hypernasality. It was expected improvement in percentage agreement with predetermined severity ratings for all evaluators, particularly for the extreme of resonances. Results showed that for the offered training, the agreement of one evaluator remained moderate after training, while there was a reduction in agreement in relation to the pre-training moment for two other evaluators, with fewer correct answers for the moderate and severe degrees. However, the two evaluators agreed with the gold standard evaluation in all speech samples with no hypernasality, in both moments, before and after training. Previous study also found better accuracy ratings for the extreme of resonance, with more stable judgments of normal resonance^(19)^. In a previous study, it was also observed that extended oral stimuli (set of oral phrases) would result in better scores of perceptive assessment reliability by expert raters, therefore, these stimuli were selected for this study^(10)^.

In this study, a single evaluator showed improvement in agreement with predetermined ratings (gold standard evaluation), with greater agreement for mild degree and absence of hypernasality. Some authors^(26)^ argued that the use of auditory anchors during rating tasks resulted in a significant improvement in the accuracy of severity ratings of speech samples in the normal and mild categories. The use of anchor samples during the training stages of this study may have also favored this single evaluator's ratings for the lower extremes of the scale, when hypernasality was absent and even mild, in which speech can be socially accepted by lay listeners^(15)^.

External variables in the speech samples may have influenced the classification of hypernasality by all evaluators for moderate and severe degrees. In this study, the presence of speech disorder characteristics (atypical articulatory patterns and nasal airflow/nasal turbulence) was not a controlled variable and, therefore, may have impaired the classification of moderate and severe hypernasality by inexperienced speech-language pathologists. Presence of dysphonia, which may affect perceptual ratings, was a controlled variable in this study.

In this study, a 4-point equal-interval scale (absent, mild, moderate, and severe) was used to rate hypernasality. This scale is widely used for clinical and research purposes^(6,10,17,19,20,27)^. In this scale, the evaluator assigns an index to the speech aspect assessed, grading the level of severity based on the assumption that the different degrees measured are equivalent to the human ear. Although the study used a 4-point scale to classify hypernasality, a dichotomous analysis of the results was further performed in order to investigate how the data would behave if the response options were only the presence or absence of hypernasality. The results showed an increase in the index of agreement with the gold standard evaluation (from significant to almost perfect) for one of the evaluators, suggesting that deciding between the presence or absence of the alteration is easier than grading the alteration, when present. A binary scale to identify hypernasality by experienced listeners was used^(28,29)^, but not to test the auditory-perceptual training effect. In further studies, the identification of the presence and absence of hypernasality could be carried out in the initial stage of a training, in order to favor the analysis of non-experienced listeners. Previous study^(22)^ offered a training that included four parts, presented in a hierarchy from simpler to more difficult tasks. This hierarchy may have favored the results, in which significant differences in the hypernasality rating scores were found between the groups receiving training (practice with and without feedback) and the group that had only exposure to speech samples.

In this study, the agreement between evaluators was also obtained in two moments: before and after training. There was significant agreement in the Cohen's kappa coefficient among the three evaluators at both times for the absent and severe degrees, suggesting that inexperienced speech-language pathologists were more consistent in their analyses, regardless of training, when hypernasality was absent or severe. The findings of this study agree with a previous study^(19)^ that also found more accurate analyzes with expertise ratings for normal and severe resonance. Although the comparison of the findings obtained before and after the auditory-perceptual training has shown an increase in the index of agreement between evaluators for absence and mild and severe hypernasality, this increase was not statistically significant. Difficulty in achieving high agreement ratings of hypernasality degree is reported in the literature, even for experienced listeners^(6,21)^.

The present study made an important contribution to a better understanding of the variables (amount of speech samples, to use anchor samples, training duration) and all of this can influence the degree`s rating of nasality made by non-expert listeners. Although it is strongly recommended, only a few studies have investigated the influence of perceptual training on rating´s agreement of non-expert listeners. The existing studies show the variability of results.

As pointed out in a recent study^(30)^, there is a need to keep searching well elaborated perceptual training for non-experts (speech language pathologist`s undergraduate students) that would enable them to perform hypernasality assessments^(30)^. This argument is based on findings of a recent study which found little or no effect of perceptual training (two hours) for identification of speech disorders presented by individuals with cleft lip and palate disorders, after offering this training for 31 students^(30)^. Findings of our study also show the need for training improvements and suggest how important it is to take care of the training offered to the future speech-language professionals.

The data found in this study shows the need for new investigation that could overcome the possible limitations. In the new studies, it is suggested to include extended training, and as often as possible, with a bigger amount of speech samples. Also, gradual increase of the severity levels of the analyses is recommended (for example, start with binary scales, and afterwards, include more amplified scales). Moreover, it is recommended to incorporate strategies that could favor identifying speech errors that may coexist with hypernasality. Offered in the beginning stages of the perceptual training, these strategies could favor posterior hypernasality ratings by the listeners. In a previous^(22)^ perceptual training strategies involving hierarchy from simpler to more difficult tasks resulted in favorable ratings by non-expert listeners. This result was not observed in other studies that did not offer easy-to-hard training procedures, as well as in the present study.

In future studies, short and long-term assessments are recommended to verify the possibility to keep the abilities learned in a long period of time^(30)^ with the offered training.

As previously proposed in the literature^(19)^, an online training can also help in the adhesion of more extensive auditory-perceptual training, by the listeners, and thus, the online modality must also be incorporated in these trainings.

## CONCLUSION

The auditory-perceptual training with controlled access to the reference samples and the immediate feedback did not result in a significant improvement in the overall percentage of correct answers obtained by the inexperienced speech-language pathologists. However, the individual data analysis has shown that the training favored one of the evaluators. Incorporating gradual and more extensive auditory-perceptual training may favor the rating task of speech hypernasality by inexperienced SLPs, particularly for mild and moderate degrees of hypernasality.
