# Supplementary material for: Effect of perceptive-auditory training on the classification of speech hypernasality
Source: Codas. 2023 Sep 15;35(6):e20220069. doi: 10.1590/2317-1782/20232022069en (PMC10723581; doi:10.1590/2317-1782/20232022069en)
Supplement: Translation

# Translation: Efeito do treinamento perceptivo-auditivo na classificação da hipernasalidade de fala

**DOI:** 10.1590/2317-1782/20232022069pt

**Authors:** Flora Taube Manicardi, Jeniffer de Cássia Rillo Dutka, Thais Alves Guerra, Maria Inês Pegoraro-Krook, Eduardo Federighi Baisi Chagas, Viviane Cristina de Castro Marino

**Affiliations:** 1 Programa de Pós-graduação em Fonoaudiologia, Universidade Estadual Paulista Júlio de Mesquita Filho - UNESP - Marília (SP), Brasil.; 2 Pós-graduação em Ciência da Reabilidação, Hospital de Reabilitação de Anomalias Craniofaciais, Universidade de São Paulo - USP -Bauru (SP), Brasil.; 3 Departamento de Educação Física, Nutrição e Fisioterapia, Universidade de Marília - Unimar - Marília (SP), Brasil.

**Keywords:** Fissura Palatina, Insuficiência Velofaríngea, Distúrbios da Fala, Percepção da Fala, Fala

## Abstract

**Objetivo:**

Analisar o efeito de um treinamento perceptivo-auditivo de fonoaudiólogas sem experiência na classificação da hipernasalidade de fala de indivíduos com fissura labiopalatina e comparar a classificação da presença e grau de hipernasalidade realizadas dessas fonoaudiólogas (com a avaliação padrão-ouro), antes e depois do treinamento perceptivo-auditivo.

**Método:**

Três fonoaudiólogas sem experiência analisaram 24 amostras de fala de alta pressão de indivíduos com fissura labiopalatina, antes e depois de treinamento perceptivo-auditivo, usando escala de quatro pontos. As amostras de fala correspondiam a seis amostras de cada grau de hipernasalidade. Entre as análises, as fonoaudiólogas receberam treinamento perceptivo-auditivo. Houve acesso às amostras de referência e feedback de respostas corretas quanto ao grau de hipernasalidade no treinamento.

**Resultados:**

Não houve diferença significativa na porcentagem geral de acertos entre os momentos antes e depois do treinamento perceptivo-auditivo. Houve associação e concordância significativa das três avaliadoras com avaliação padrão ouro após treinamento, com aumento da concordância para uma avaliadora (aumento de respostas corretas para os graus ausente e leve). A análise dicotômica dos dados mostrou aumento do índice de concordância Kappa dessa avaliadora. Houve aumento do índice concordância inter-avaliadores para hipernasalidade ausente, leve, e grave, porém sem significância estatística.

**Conclusão:**

O treinamento perceptivo-auditivo não resultou em melhora significativa da classificação da hipernasalidade de fala pelas fonoaudiólogas sem experiência, embora a análise individual dos dados tenha mostrado que o treinamento favoreceu uma dessas avaliadoras. Novos estudos envolvendo treinamento perceptivo-auditivo gradual e mais extenso poderão favorecer a classificação da hipernasalidade de fala por fonoaudiólogos sem experiência.

## INTRODUÇÃO

A hipernasalidade de fala é considerada o principal sintoma da disfunção velofaríngea (DVF) após correção cirúrgica primária da fissura labiopalatina (FLP)^(1)^. Esse excesso de ressonância nasal presente durante a produção dos sons orais^(1)^ ocorre devido o acoplamento das cavidades de ressonância (oral e nasal). Embora medidas instrumentais (nasoendoscopia, videofluoroscopia, nasometria e técnica de fluxo-pressão) sejam recomendadas para corroborar com o diagnóstico de DVF, a avaliação perceptiva é a ferramenta inicial utilizada pelos clínicos para identificar os sintomas de fala sugestivos da DVF após palatoplastia primária^(2,3)^. Por meio de suas impressões auditivas, os clínicos identificam a presença da hipernasalidade e aferem sua gravidade, o que favorece a tomada de decisões clínicas e a avaliação da efetividade do tratamento^(4)^. A avaliação perceptivo-auditiva deve ser realizada por profissionais experientes, a fim de minimizar variações e erros inerentes à natureza subjetiva deste tipo de avaliação^(5)^.

A identificação da hipernasalidade pode ser desafiadora mesmo para ouvintes experientes^(6,7)^. O tipo de estímulo (fala ou canto)^(8)^, a extensão do estímulo de fala, o contexto fonético da amostra de fala^(1,9,10)^ e a presença de alterações co-existentes^(11)^ podem influenciar na avaliação perceptiva da hipernasalidade, afetando sua confiabilidade^(4)^. O tipo de escala^(4,12-14)^ e a familiaridade do ouvinte com o uso de uma determinada escala também podem influenciar na avaliação desses sintomas de fala^(13)^. A escala numérica de intervalos iguais é amplamente utilizada para fins clínicos^(15)^ e em pesquisas^(6,10,16-18)^, incluindo aquelas envolvendo treinamento perceptivo-auditivo para classificação da hipernasalidade^(19,20)^.

O grau de experiência clínica e os critérios adotados por cada avaliador em suas análises também pode afetar a classificação da hipernasalidade^(4)^. O treinamento perceptivo-auditivo e o uso de amostras de referências são considerados estratégias que podem favorecer a consistência das análises de avaliadores^(6)^, em que padrões internos instáveis dos ouvintes podem ser substituídos por referências^(4)^. Embora essas estratégias sejam fortemente recomendadas para melhorar a confiabilidade da análise perceptivo-auditivo da hipernasalidade^(6,21)^, poucos estudos, documentaram os resultados do treinamento perceptivo-auditivo na avaliação desse sintoma de fala^(6,19-21)^.

Em estudo anterior^(6)^, o treinamento controlado e a definição de critérios padronizados para a classificação da hipernasalidade levaram ao aumento de índice de concordância intra e interavaliadores e, consequentemente, melhora na confiabilidade da análise perceptivo-auditiva deste sintoma de fala. Outros estudos investigaram particularmente a influência do treinamento perceptivo-auditivo na análise da hipernasalidade realizados por estudantes de fonoaudiologia^(20,22)^ ou residentes em otorrinolaringologia^(19)^.

Lee et al.^(22)^, estudaram o efeito do treinamento e do *feedback* na confiabilidade intra e interavaliadores durante a análise da hipernasalidade realizada por estudantes de fonoaudiologia. Foi encontrada diferença significativa entre (1) os grupos que receberam treinamento (treinamento com e sem *feedback*) e (2) o grupo que teve apenas exposição das amostras de fala. Os grupos que receberam treinamento mais abrangente tiveram melhora significativa na classificação da hipernasalidade quando comparado ao grupo que teve apenas exposição das amostras. Não houve diferença significativa na confiabilidade intra-avaliadores entre os três grupos de ouvintes (treinamento com feedback, treinamento sem feedback e exposição das amostras). Os autores atribuíram este resultado ao pequeno número de amostras (82 amostras divididas nas 4 etapas do treinamento) e duração limitada do treinamento (30 minutos de introdução e uma hora em cada etapa do treinamento), o que pode ter influenciado para que os ouvintes atingissem boa confiabilidade.

Em estudo posterior, Guerra^(20)^ investigou a classificação da hipernasalidade realizada por estudantes de graduação em Fonoaudiologia. As análises foram feitas em diferentes tempos (antes do treinamento, imediatamente após treinamento, uma semana e um mês após treinamento) e em quatro condições distintas: sem treinamento, com treinamento e acesso opcional às amostras de referência; com treinamento e acesso controlado às amostras de referência; e, com treinamento, acesso controlado às amostras de referência e *feedback* imediato de resposta correta. Não foi encontrada diferença significativa nos resultados da classificação da hipernasalidade nas três modalidades de treinamento e nos diferentes tempos de análise, mesmo controlando o possível impacto de alterações articulatórias na classificação da hipernasalidade, ao usar somente amostras de fala de baixa pressão intra-oral.

Sydney et al.^(19)^ determinaram a capacidade de residentes de otorrinolaringologia (grupo controle vs. grupo treinamento) em avaliar a hipernasalidade de fala em pacientes com DVF. Embora tenha havido uma melhora na concordância após treinamento, essa diferença não foi significativa.

Há uma necessidade de melhor compreensão da capacidade de avaliação da hipernasalidade por avaliadores sem experiência, bem como estratégias que podem melhorar suas classificações iniciais. Achados prévios^(20,22)^ envolvendo análises perceptivo-auditiva realizadas por estudantes em Fonoaudiologia são inconsistentes na literatura atual. Além disso, para os residentes em otorrinolaringologia, houve tendência de melhora na concordância das respostas após treinamento, porém sem diferença significativa^(19)^. Nesse sentido, não é claro se um treinamento perceptivo-auditivo controlado pode influenciar a classificação da hipernasalidade por ouvintes com limitada experiência clínica na área da Fonoaudiologia.

O objetivo do estudo foi (1) analisar o efeito de um treinamento perceptivo-auditivo de fonoaudiólogas sem experiência na classificação da hipernasalidade de fala de indivíduos com FLP e, (2) comparar a classificação da presença e grau de hipernasalidade dessas fonoaudiólogas com a avaliação padrão-ouro, antes e depois de treinamento perceptivo-auditivo.

## MÉTODO

O estudo foi aprovado pelo Comitê de Ética em Pesquisa com Seres Humanos do Hospital de Reabilitação de Anomalias Craniofaciais - Universidade de São Paulo, Bauru, SP, Brasil (parecer nº 3.131.704). Todos os participantes que concordaram em participar do estudo assinaram o Termo de Consentimento Livre e Esclarecido (TCLE).

Foram selecionadas gravações de fala pré-existentes de indivíduos com histórico de fissura labiopalatina (FLP), de ambos os sexos. As gravações de fala pré-existentes selecionadas foram avaliadas posteriormente por três participantes, em dois momentos: antes e depois do treinamento perceptivo-auditivo.

### Amostras de fala

As gravações de fala foram selecionadas de um conjunto de gravações pré-existente em uma base de dados. Essas gravações foram originalmente obtidas em uma sala acusticamente tratada e tinham boa qualidade de áudio. O material foi gravado diretamente do computador, equipado com placa de som *Sound Blaster Audigy 2* e programa Sony® Sound Forge, versão 8.0, com taxa de amostragem de 44100 Hz, em monocanal, 16 Bits. O sinal de áudio foi capturado utilizando microfone de cabeça (modelo AKG C420®). Todas as gravações pré-existentes tiveram um intervalo padronizado de um segundo entre cada frase. O estímulo de fala do estudo compreendeu um conjunto de 12 frases de alta pressão intraoral, em que cada frase era composta por um único som-alvo em recorrência. Este estímulo foi selecionado considerando os achados de estudo prévio que mostrou análises perceptivas da hipernasalidade mais favoráveis para estímulos orais mais extensos, mesmo na presença de alterações de fala coexistentes.

As amostras incluídas no estudo compreenderam 48 amostras de fala (em áudio) armazenadas na base de dados, que eram representativas dos graus de hipernasalidade: ausente (A), leve (HL), moderado (HM) e grave (HG), nas vozes masculinas e femininas. Estas amostras eram constituídas por um conjunto de 12 frases de alta pressão. Para fins de seleção, foram ouvidas as amostras de fala da base de dados e as primeiras 12 amostras desta base representativas de cada um dos 4 graus de hipernasalidade foram incluídas, totalizando 48 amostras. A representatividade das amostras de fala gravadas quantos aos quatro graus da escala (hipernasalidade A, 25% das gravações; HL, 25% das gravações, HM, 25% das gravações e HG, 25% das gravações) foi estabelecida em estudo prévio^(23)^.

No estudo prévio de Silva-Mori^(23)^ três fonoaudiólogos com experiência de pelo menos 10 anos em um centro craniofacial e com rotina clínica diária na identificação de distúrbios de fala em indivíduos com fissura labiopalatina, obtiveram 100% de concordância em suas avaliações. Portanto, para o atual estudo, as análises das 48 amostras de fala feitas por esses fonoaudiólogos especialistas foram usadas como avaliação padrão-ouro para comparações com as avaliações dos participantes e, também, para selecionar as amostras de fala do treinamento perceptivo-auditivo. As amostras de fala incluídas neste estudo foram controladas para disfonia. Outros sintomas de fala (emissão de ar nasal/turbulência, articulação compensatória) não foram controlados.

As 48 amostras de fala representativas dos quatro graus de hipernasalidade inicialmente selecionadas para o estudo foram posteriormente distribuídas em amostras de classificação, referência, treinamento e retreinamento. Assim, as 48 amostras foram distribuídas em: 24 amostras de classificação (análises das avaliadoras, antes e depois do treinamento), 8 amostras de referência (âncoras), 8 amostras de treinamento perceptivo-auditivo inicial e 8 amostras de retreinamento perceptivo-auditivo. Por sua vez, as 24 amostras de classificação eram constituídas por 6 amostras representativas de cada 4 graus de hipernasalidade (6 A, 6 HL, 6 HM, 6 HG), pareadas por sexo. Por fim, as 8 amostras de referência, 8 amostras de treinamento e 8 amostras de retreinamento eram constituídas por 2 amostras de cada grau de hipernasalidade (2 A, 2 HL, 2 HM, 2 HG), também pareadas por sexo.

### Participantes

Três fonoaudiólogas certificadas, com idade média de 24 anos, falantes nativas do Português Brasileiro, foram convidadas para participar desse estudo. Todas as participantes estavam iniciando atividades em um programa de residência multiprofissional em saúde na área de anomalias craniofaciais. Todas as participantes relataram ter audição normal e nenhuma experiência anterior na classificação dos graus de hipernasalidade em indivíduos com FLP.

### Procedimentos

As participantes analisaram, por meio da escala de 4 pontos, as 24 amostras de classificação (6A, 6HL, 6HM, 6HG) em dois momentos: antes e depois de um treinamento perceptivo-auditivo. As amostras de fala foram apresentadas simultaneamente pela primeira autora (FTM), por meio de um divisor de sinal do tipo Powerplay PRO-8 HA8000, fones de ouvido individuais de alta qualidade (AKG Harman, modelo K414P) e as respostas foram inseridas em uma planilha Excel.

As amostras puderam ser ouvidas quantas vezes cada participante julgasse necessário para realizar seu julgamento. Quando havia a necessidade, a participante solicitava a repetição da amostra para avaliadora (primeira autora) e as outras participantes escolhiam escutar novamente ou não. Outros sintomas de fala (emissão de ar nasal, articulação compensatória) não foram controlados e apenas amostras com voz normal foram incluídas neste estudo.

#### Treinamento perceptivo-auditivo

O treinamento perceptivo-auditivo foi oferecido por meio de uma apresentação em *PowerPoint* e foi constituído de uma sessão de calibração, treinamento inicial e retreinamento. Na sessão de calibração, foram apresentados às participantes o conceito de hipernasalidade e foram dadas orientações sobre o uso da escala de 4 pontos para classificação dos graus da hipernasalidade. Três amostras de fala gravadas (não incluídas nas demais etapas do estudo) foram apresentadas às participantes para familiarização da tarefa.

O treinamento inicial e de retreinamento foram oferecidos pela primeira autora do estudo, de forma presencial, para cada participante. Este realizou o treinamento e retreinamento individualmente, utilizando fone de ouvido de alta qualidade (AKG Harman, modelo K414P) e um computador para indicar suas respostas em planilha Excel^®^.

Para o treinamento perceptivo-auditivo propriamente dito, foram apresentadas a cada participante oito amostras de referências (amostras âncora), sendo quatro vozes masculinas e quatro vozes femininas, representando cada grau de hipernasalidade (A, HL, HM, HG). As participantes foram instruídas a avaliar a hipernasalidade por meio de uma escala de 4 pontos. Após cada resposta, a pesquisadora forneceu *feedback* de resposta correta quanto ao grau de hipernasalidade. Caso a participante avaliasse a amostra incorretamente, a pesquisadora reapresentava duas amostras de referência. Uma amostra de referência correspondia à classificação correta do grau de hipernasalidade e a outra amostra de referência correspondia à classificação da avaliadora (incorreta). Após, a pesquisadora apresentava novamente a amostra-alvo apenas para possibilitar a comparação, pela participante, desta amostra-alvo com as duas amostras de referências. A cada 20 minutos de treinamento, foi realizado um intervalo de cinco minutos para evitar fadiga.

O retreinamento foi realizado uma semana após o treinamento perceptivo-auditivo inicial, utilizando 8 amostras de fala distintas daquelas usadas no treinamento. As mesmas 8 amostras de referência do treinamento perceptivo-auditivo inicial foram utilizadas no retreinamento. Os procedimentos utilizados no treinamento perceptivo-auditivo inicial foram adotados no retreinamento, com uma única diferença: a ordem de apresentação das amostras que no retreinamento foi aleatorizada, a fim de preparar as participantes para a etapa de classificação final.

### Forma de análise dos dados

As variáveis quantitativas estão descritas pela média e desvio-padrão (DP). A distribuição de normalidade da porcentagem de acertos foi verificada pelo teste de Kolmogorov-Smirnov. Para comparação da média de porcentagem de acertos das respostas das participantes em relação a avaliação padrão-ouro, nos momentos antes e depois do treinamento perceptivo-auditivo, foi utilizado o teste não paramétrico Wilcoxon.

As variáveis qualitativas foram descritas pela distribuição de frequência absoluta. A associação entre variáveis qualitativas (respostas de cada participante versus avaliação padrão-ouro) foi analisada por meio do teste do Qui-quadrado para associação, levando em conta os graus de hipernasalidade. O teste Qui-quadrado também foi usado para verificar a associação entre as variáveis qualitativas presença e ausência da hipernasalidade (análise dicotômica), nos dois momentos, antes e depois do treinamento perceptivo-auditivo.

O coeficiente Kappa (k) também foi obtido para análise da concordância entre cada participante e avaliação padrão-ouro por momento. Os valores do coeficiente Kappa (k), foram interpretados em: abaixo de 0 sem concordância; de 0 a 0,19 concordância pobre; de 0,20 a 0,39 concordância regular; de 0,40 a 0,59 concordância moderada; de 0,60 a 0,79 concordância substancial; de 0,80 a 1,00 concordância quase perfeita). A diferença entre os momentos (antes e depois do treinamento) para o k foram analisados pelos intervalos de confiança 95%. Para todas as análises foi utilizado o software SPSS versão 19.0 for Windows, sendo adotado nível de significância de 5% (p<0,05).

## RESULTADOS

A média da porcentagem de acertos da classificação do grau de hipernasalidade das três participantes (avaliadoras), nos momentos antes e depois do treinamento perceptivo-auditivo foi de 65,3% e 62,5%, respectivamente. Não houve diferença significativa na porcentagem geral de acertos entre os momentos antes e depois do treinamento perceptivo-auditivo (p-valor=0,972; teste não paramétrico de Wilcoxon).

Além disso, antes do treinamento, foi observada associação significativa das três avaliadoras (AV1, AV2 e AV3) com a avaliação padrão-ouro. A AV1 apresentou concordância moderada, a AV2 concordância substancial e a AV3 concordância regular (Tabela 1). No momento depois treinamento, foi observada associação e concordância significativa das três avaliadoras (AV1, AV2 e AV3) com a avaliação padrão-ouro, porém a AV1 continuou apresentando concordância moderada, a AV2 teve redução na concordância em relação ao momento pré-treinamento e a AV3 apresentou melhora na concordância (Tabela 2).

**Tabela 1:** Análise da concordância e associação da distribuição de frequência absoluta da avaliação da classificação da hipernasalidade de cada avaliador com a avaliação padrão-ouro, antes do treinamento perceptivo-auditivo, por grau de hipernasalidade^(1)^

Antes do Treinamento		Avaliação Padrão-Ouro				*X^2^*	
		Ausente	Leve	Moderada	Grave	p-valor	Kappa
AV 1	Ausente	**6**	2	0	0	<0,001*	0,556‡
	Leve	0	**4**	3	0		
	Moderada	0	0	**3**	3		
	Grave	0	0	0	**3**		
AV 2	Ausente	**6**	2	0	0	<0,001*	0,667‡
	Leve	0	**3**	1	1		
	Moderada	0	0	**5**	1		
	Grave	0	1	0	**4**		
AV 3	Ausente	**3**	2	0	0	<0,001*	0,389
	Leve	3	**2**	1	1		
	Moderada	0	2	**5**	2		
	Grave	0	0	0	**3**		

* associação significativa pelo teste do Qui-quadrado para p-valor ≤0,05;

‡ coeficiente kappa significativo para p-valor ≤0,05. Achados em negrito significam resultados que concordam com a avaliação padrão-ouro

**Legenda:** AV=avaliador

**Tabela 2:** Análise da concordância e associação da distribuição de frequência absoluta da avaliação da classificação da hipernasalidade de cada avaliador com a avaliação padrão-ouro, depois do treinamento perceptivo-auditivo, por grau de hipernasalidade^(2)^

Depois do Treinamento		Avaliação Padrão-Ouro				*X^2^*	Kappa
		Ausente	Leve	Moderada	Grave	p-valor	*K*
AV 1	Ausente	**6**	1	0	1	<0,001*	0,500‡
	Leve	0	**5**	2	0		
	Moderada	0	0	**2**	3		
	Grave	0	0	2	**2**		
AV 2	Ausente	**6**	2	0	0	<0,001*	0,333‡
	Leve	0	**3**	3	2		
	Moderada	0	1	**1**	2		
	Grave	0	0	2	**2**		
AV 3	Ausente	**6**	1	0	0	<0,001*	0,667‡
	Leve	0	**5**	1	1		
	Moderada	0	0	**4**	2		
	Grave	0	0	1	**3**		

* associação significativa pelo teste do Qui-quadrado para p-valor ≤0,05;

‡ coeficiente kappa significativo para p-valor ≤0,05. Achados em negrito significam resultados que concordam com a avaliação padrão-ouro

**Legenda:** AV=avaliador

A análise dicotômica (presença e ausência de hipernasalidade) mostrou que, antes do treinamento perceptivo-auditivo, todas as avaliadoras apresentaram associação significativa e concordância significativa com a avaliação padrão-ouro. No entanto, depois do treinamento perceptivo-auditivo, a AV3 apresentou aumento do índice de concordância Kappa com a avaliação padrão-ouro (Tabela 3)

**Tabela 3:** Comparação dos resultados dos participantes (avaliadores) com a avaliação padrão-ouro, antes e depois do treinamento perceptivo-auditivo, de acordo com a análise dicotômica^(24)^

		Avaliação Padrão-Ouro		*X^2^ p valor*	Kappa
		Presente	Ausente		
Antes do treinamento					
AV 1	Presente	**16**	0	<0,001*	0,800‡
	Ausente	2	6		
AV 2	Presente	**16**	0	<0,001*	0,800‡
	Ausente	2	6		
AV 3	Presente	**16**	3	0,047*	0,412‡
	Ausente	2	3		
Depois do treinamento					
AV1	Presente	**16**	0	<0,001*	0,800‡
	Ausente	2	6		
AV 2	Presente	**16**	0	<0,001*	0,800‡
	Ausente	2	6		
AV 3	Presente	**17**	0	<0,001*	0,895‡
	Ausente	1	6		

* associação significativa pelo teste do Qui-quadrado para p-valor ≤0,05;

‡ coeficiente kappa significativo para p-valor ≤0,05

**Legenda:** AV=avaliador.

A tabela 4 mostra os resultados da análise da concordância entre as avaliadoras, nos momentos antes e depois do treinamento perceptivo-auditivo, por grau de hipernasalidade e classificações totais. Embora, antes do treinamento perceptivo-auditivo, tenha sido encontrada concordância significativa no coeficiente Kappa entre as três avaliadoras para os graus ausente, moderado, grave e no total, não houve concordância entre as avaliadoras para a hipernasalidade de grau leve. Além disso, depois do treinamento perceptivo-auditivo, houve concordância significativa no coeficiente Kappa entre as avaliadoras para os graus de hipernasalidade ausente, leve, grave e total, porém sem concordância para hipernasalidade moderada. Ao comparar os achados obtidos antes e depois do treinamento perceptivo-auditivo, foi possível notar aumento do índice concordância para hipernasalidade ausente, leve, grave e no total, porém este aumento não teve significância estatística (análise do intervalo de confiança de 95%).

**Tabela 4:** Concordância entre os avaliadores (coeficiente Kappa) antes e depois do treinamento perceptivo-auditivo por categoria de resposta e para concordância total^(3)^

Treinamento	Categorias	Kappa	IC 95%		p-valor
			LI	LS	
Antes	hipernasalidade ausente	0,597	0,366	0,828	<0,001*
	hipernasalidade leve	-0,072	-0,303	0,158	0,538
	hipernasalidade moderada	0,261	0,030	0,491	0,027*
	hipernasalidade grave	0,356	0,125	0,587	0,003*
	Total	0,284	0,147	0,420	<0,001*
Depois	hipernasalidade ausente	0,681	0,450	0,912	<0,001*
	hipernasalidade leve	0,280	0,049	0,511	0,0018*
	hipernasalidade moderada	0,074	-0,157	0,305	0,532
	hipernasalidade grave	0,600	0,369	0,831	<0,001*
	Total	0,413	0,276	0,550	<0,001*

* coeficiente kappa significativo para p-valor ≤0,05

**Legenda:** IC = Intervalo de Confiança; LI = Limite Interior; LS = Limite Superior

## DISCUSSÃO

Este estudo investigou o efeito do treinamento perceptivo-auditivo de fonoaudiólogos sem experiência na classificação da hipernasalidade de fala de indivíduos com FLP. Os resultados obtidos no presente estudo concordam com o estudo de Sydney et al.^(19)^, que demonstrou que o treinamento não melhorou a tarefa de classificação das amostras de fala pelos participantes.

Os achados do presente estudo divergem dos resultados obtidos por Lee et al.^(22)^. Os autores encontraram uma diferença significativa nos escores de análise de hipernasalidade para os grupos que receberam treinamento (com ou sem *feedback*) e o grupo que teve somente exposição às amostras de fala. Embora o presente estudo também tenha oferecido treinamento e retreinamento, ambos com *feedback* para respostas corretas, o foco do treinamento foi apenas a classificação dos graus de hipernasalidade, independentemente desse sintoma ocorrer em coexistência com outras alterações de fala. No estudo de Lee et al.^(22)^, por outro lado, foi oferecido um treinamento gradual, em uma hierarquia de tarefas mais simples às mais difíceis, o que pode ter favorecido a avaliação da hipernasalidade mesmo na coexistência de outras alterações de fala.

Os achados do presente estudo concordam com os obtidos anteriormente por Guerra^(20)^. Alguns pesquisadores argumentam que avaliar o grau de hipernasalidade pode ser ainda mais desafiador com a presença de múltiplas alterações de fala devido à necessidade de isolar com precisão as características perceptivo-auditivas de distintas alterações de fala durante a análise^(25)^. Guerra^(20)^ controlou o possível impacto das articulações compensatórias nas avaliações da hipernasalidade realizadas por alunos de fonoaudiologia, utilizando apenas amostras de fala com sons de baixa pressão intra-oral. O presente estudo, no entanto, controlou apenas alterações de voz, sugerindo a necessidade de novas propostas de treinamento. Futuros estudos, portanto, são recomendados visando-se ampliar o treinamento dos avaliadores para também capacitá-los a identificar outras alterações de fala na presença e ausência de hipernasalidade,

O presente estudo também analisou a associação entre variáveis qualitativas (graus de hipernasalidade), sendo que se esperava uma melhora na porcentagem da concordância dos avaliadores participantes em relação à avaliação padrão-ouro, principalmente nos extremos da escala. Os resultados revelaram que para o treinamento oferecido a concordância de uma avaliadora permaneceu moderada depois do treinamento enquanto para as outras duas avaliadoras houve redução na concordância em relação ao momento pré-treinamento, com menor quantidade de respostas corretas para os graus moderado e grave. Essas duas avaliadoras, porém, concordaram com avaliação padrão-ouro em todas as amostras de fala que apresentavam ausência da hipernasalidade, em ambos os momentos, antes e depois do treinamento. Esses achados concordam com estudo prévio na qual foi observada melhor acuracidade nas análises para o extremo da escala, particularmente quando a fala era normal^(19)^. Em estudo prévio, também foi observado que estímulos orais de maior extensão (conjunto de frases orais) resultaram em melhor índice de confiabilidade em análises perceptivas de avaliadores experientes e, portanto, estes estímulos foram selecionados para o presente estudo^(10)^.

Uma única avaliadora do presente estudo apresentou melhora na concordância em relação ao padrão-ouro, com maior concordância para o grau leve e, também, para a ausência da hipernasalidade. Alguns autores^(26)^ argumentam que o uso de amostras âncoras durante a tarefa de classificação resulta em melhora significativa na acurácia das classificações de gravidade das amostras de fala nos graus normal e leve. O uso de amostras âncoras durante a etapa de treinamento desse estudo pode ter favorecido a avaliação dessa única avaliadora quando a hipernasalidade era ausente ou leve, ou seja, onde a fala pode ser socialmente aceita por ouvintes leigos^(15)^.

Fatores externos às amostras de fala podem ter influenciado na classificação da hipernasalidade por todas as avaliadoras para os graus moderado e severo. Neste estudo, a presença de padrões articulatórios atípicos e o escape de ar audível/ronco nasal, por um lado, não foram variáveis controladas e podem ter desfavorecido a classificação da hipernasalidade dos graus moderado e grave pelas fonoaudiólogas sem experiência. Por outro lado, outra variável que pode afetar análises perceptivas é a presença de disfonia^(1)^ e, por esse motivo, essa variável foi controlada nesse estudo.

O método utilizado para classificação da hipernasalidade no estudo foi a escala de intervalos iguais de 4 pontos (ausente, leve, moderado e grave). Esta escala é amplamente utilizada para fins clínicos e de pesquisa^(6,10,17,19,20,27)^. Nesta escala, o avaliador atribui um índice ao aspecto de fala avaliado, graduando o nível de gravidade partindo do pressuposto que os diferentes graus aferidos são equivalentes para a orelha humana. Embora 4 pontos para a classificação da hipernasalidade, a análise de forma dicotômica dos resultados foi realizada, a fim de verificar como os dados se comportariam se as opções de respostas fossem somente presença ou ausência da hipernasalidade. Os resultados mostraram aumento do índice de concordância com a avaliação padrão-ouro (de substancial para quase perfeito) para uma das avaliadoras, confirmando que decidir entre a presença ou ausência da alteração é mais fácil do que a graduar a alteração, quando presente. A escala binária para identificar a hipernasalidade por ouvintes experientes já foi utilizada anteriormente^(28,29)^, porém para testar o efeito de treinamento perceptivo-auditivo. Em estudos futuros, a identificação da presença e ausência da hipernasalidade poderia ser realizada na fase inicial de um treinamento, a fim de favorecer a análise de ouvintes sem experiência. Estudo anterior^(22)^ ofereceu um treinamento que incluía quatro partes, apresentando uma hierarquia de tarefas mais simples às mais complexas. Essa hierarquia pode ter favorecido os resultados, nos quais foram encontradas diferenças significativas nos escores de classificação da hipernasalidade entre os grupos que receberam treinamento (prática com e sem feedback) e o grupo que teve apenas exposição a amostras de fala.

A concordância entre avaliadores foi obtida neste estudo em dois momentos: antes e depois do treinamento. Houve concordância significativa no coeficiente Kappa entre as três avaliadoras, em ambos os momentos, para os graus ausente e grave, sugerindo que fonoaudiólogos sem experiência foram mais consistentes em suas análises, independentemente de treinamento, quando a hipernasalidade era ausente ou grave. Este achado corrobora com estudo prévio^(19)^ em que também foram verificadas análises mais acuradas (em relação ao padrão-ouro) para fala normal e grave. Embora a comparação dos achados obtidos antes e depois do treinamento perceptivo-auditivo mostrou aumento do índice concordância entre avaliadoras para hipernasalidade ausente, leve e grave, este aumento não teve significância estatística. A dificuldade em atingir altos índices de concordância na classificação da hipernasalidade é reportada na literatura, mesmo para ouvintes com experiência^(6,21)^.

O presente estudo traz contribuições importantes para um maior entendimento sobre variáveis (quantidade de amostras de fala, uso de amostras de referência, duração de treinamento) que podem influenciar a classificação dos graus de nasalidade de fala por ouvintes não treinados. Embora fortemente recomendado, poucos estudos investigaram a influência do treinamento perceptivo na confiabilidade das análises de amostras de fala de ouvintes não treinados e os estudos existentes mostram variabilidade nos resultados. Conforme destacado em estudo recente^(30)^, há necessidade em se dar continuidade na busca de treinamentos perceptivo-auditivos estruturados direcionados à ouvintes não experientes (alunos de cursos de fonoaudiologia) que possam capacitá-los a realizar análises confiáveis da hipernasalidade de fala^(30)^. Tal argumento é fundamentado nos achados desse estudo recente em que pouco ou nenhum efeito foi encontrado após treinamento perceptivo (duas horas) direcionados às alterações de fala de indivíduos com fissura labiopalatina oferecidos a 31 alunos em formação^(30)^. Ou seja, os achados do presente estudo também apontam para a necessidade do aprimoramento do treinamento e sugerem a importância do cuidado diante dos treinamentos oferecidos durante a formação de futuros fonoaudiólogos.

Os achados obtidos no estudo apontam para a necessidade de novas investigações que possam superar possíveis limitações. Em estudos futuros sugere-se incluir treinamentos mais extensos e, sempre que possível, com maior quantidade de amostras, e com aumento gradativo de níveis de dificuldades nas análises (por exemplo, iniciando com escalas binárias e, posteriormente, incluindo escalas mais amplas). Além disso, recomenda-se incorporar estratégias que permitam identificar os erros de fala que podem coexistir com a hipernasalidade de fala, nos estágios iniciais do treinamento perceptivo-auditivo, o que pode favorecer a posterior classificação da hipernasalidade de fala pelos participantes. Em estudo prévio, estratégias de treinamento com aumento gradativo de níveis de dificuldades nas análises resultaram em análises favoráveis pelos ouvintes não experientes, fato pouco ou não observado em outros estudos que não utilizaram estas estratégias, incluindo a presente pesquisa.

Em estudos futuros, análises de avaliações realizadas imediatamente e a longo prazo são recomendadas, a fim de verificar a possibilidade de manutenção das habilidades aprendidas à longo prazo^(30)^ com os treinamentos oferecidos

Conforme proposto anteriormente na literatura^(19)^ um treinamento online também pode auxiliar na adesão de treinamentos perceptivo-auditivo mais extensos por parte dos avaliadores e, assim, a modalidade online também deve ser incorporada nestes treinamentos.

## CONCLUSÃO

O treinamento perceptivo-auditivo com acesso controlado às amostras de referência e o *feedback*de resposta correta não resultou em melhora significativa na porcentagem geral de acertos obtidas pelas fonoaudiólogas sem experiência, embora a análise individual dos dados tenha mostrado que o treinamento favoreceu uma das avaliadoras. A incorporação de um treinamento perceptivo-auditivo gradual e mais extenso pode favorecer a tarefa de classificação da hipernasalidade de fala por fonoaudiólogos sem experiência, principalmente para os graus de hipernasalidade leve e moderado.
